# Research highlights, *The Plant Genome*, Volume 15, Issue 3

**DOI:** 10.1002/tpg2.20256

**Published:** 2022-09-07

## 1 EVOLUTIONARY FATES OF GENE‐BODY METHYLATION AND ITS DIVERGENT ASSOCIATION WITH GENE EXPRESSION IN PIGEONPEA

Pigeonpea is an important legume crop that is grown and consumed worldwide. Junaid et al. (https://doi.org/10.1002/tpg2.20207) investigate the patterns of gene body methylation (GbM) in three different genotypes of pigeonpea (11A male sterile, 303 fertile, and their fertile F1 hybrid). Their findings identify conserved methylation patterns in genes associated with nitrogen transport and its metabolism related genes during evolution of legumes. These may be promising candidates for generation of epialleles for developing pigeonpea cultivars with high protein content without modifying their DNA sequence. They also found that transposons influence methylation and expression patterns of genes that originated from different modes of duplication. The study also provides advances on understanding of how the epigenetic mechanisms substantiate duplicated gene retention and neofunctionalization during evolution. Also, this work is timely because substantiating efforts toward developing high yielding crops is the need of the hour to meet the challenges of rising global hunger.

## 2 COMPARING GRADIENT BOOSTING MACHINE AND BAYESIAN THRESHOLD BLUP FOR GENOME‐BASED PREDICTION OF CATEGORICAL TRAITS IN WHEAT BREEDING

Genomic selection trains a statistical machine‐learning model using available phenotypic and genotypic data with which predictions are performed for individuals that were only genotyped. The exploration of new statistical machine‐learning algorithms is necessary for increasing genome‐based prediction accuracy. In this paper, Montesinos‐López et al. (https://doi.org/10.1002/tpg2.20214) compared the genomic‐enabled prediction accuracy of the Bayesian threshold genomic best linear unbiased predictor model (TGBLUP) and the gradient boosting machine (GBM) using four real wheat data sets with categorical traits. We compared the two algorithms and found that in three of the four data sets, the GBM outperformed the TGBLUP model. In the larger data sets (Data Sets 3 and 4), the gain in terms of prediction accuracy of the GBM was considerably significant.

## 3 QUANTITATIVE TRAIT LOCI IN WHEAT THAT GIVE DURABLE LEAF RESISTANCE

New races of leaf rust, caused by *Puccinia triticina*, with virulence to important resistance genes in wheat are continuously emerging. The increase and spread of new virulent races often results in the reduction of leaf rust resistance in widely planted wheat cultivars. The wheat breeding line CI13227 has durable leaf rust resistance, and Kolmer and Rouse (https://doi.org/10.1002/tpg2.20215) characterized three quantitative trait loci derived from CI13227 that when combined conditioned high levels of resistance in field plot tests. The quantitative trait loci are effective in the adult plant stage only and gave resistance to a mixture of current and past *P. triticina* races. DNA markers for the quantitative trait loci were developed to facilitate marker‐based selection in breeding programs. The resistance derived from CI13227 can be used to improve leaf rust resistance in wheat.

## 4 MAPKS VARY UNDER DROUGHT

MAPK gene families are important for various plant physiological processes. Shi et al. (https://doi.org/10.1002/tpg2.20216) reported the whole genome identification and characterization of three MAPK families from a maize inbred line Huangzaosi, which exhibit more robust drought tolerance than B73. Genes with high basal expression levels in root tissues were selected to reveal their response to drought conditions, and the differential expression profiles of certain members may contribute to distinct drought tolerance at different developmental stages of the two lines.

## 5 THE INDIAN MANGROVE GENOME REVEALED A GENOME DUPLICATION AND THE POPULATION STRUCTURE

Mangrove forests can provide protection to coastlines and habitats for marine species. However, these valuable ecosystems are declining at an alarming rate because of overexploitation and coastal development. Pootakham et al. (https://doi.org/10.1002/tpg2.20217) report the reference genome of Indian mangrove and revealed that a genome‐wide duplication event occurred before the divergence of Rhizophoraceae mangrove species. The genome sequence was used as a reference for mapping short reads to assess genetic diversity of the C. tagal population. The population structure of C. tagal was the admixture of the 2 subpopulations influenced by location: one from the Andaman coast and the other from the Gulf of Thailand coast.

## 6 RICE GENOMIC SELECTION MARKER SET

Genomic selection is a powerful breeding approach where genotypic information is used to predict the future performance of untested breeding lines. This technology requires a cost‐effective method to genotype the material developed in the breeding program. Cerioli et al. (https://doi.org/10.1002/tpg2.20219) designed and developed a marker set optimized for routine genotyping and genomic selection applications in the southern U.S. rice germplasm. The marker set was validated using multiple years of phenotype data within and across breeding populations. Cross validation demonstrated good predictive ability for yield, grain quality, and agronomic traits. Comparison to phenotypic selection highlights the potential of genomic selection applications in an applied rice breeding context. The marker set is available for outsourcing through the genotyping service provider Agriplex.

## 7 WHEAT PANACHE: A PANGENOME GRAPH DATABASE REPRESENTING PRESENCE‐ABSENCE VARIATION ACROSS SIXTEEN BREAD WHEAT GENOTYPES

Bread wheat is one of humanity's staple foods, but climate change threatens wheat yields. Genomics methods can accelerate the breeding of advanced wheat varieties, but the wheat genome is very large, complex, and differs significantly between wheat varieties. Bayer et al. (https://doi.org/10.1002/tpg2.20221) present a wheat graph pangenome combining the assemblies of 16 bread wheat cultivars. This graph show that only 65% of the genomic areas are present in all 16 cultivars, with several agronomically important genes being highly variable. This graph is visualized in a wheat Panache database, through a website that allows users search the assembly for regions of interest. The graph pangenome and wheat Panache will help breeders and researchers identify variation in the genome responsible for important traits.

## 8 IDENTIFICATION OF FHB RESISTANCE IN WHEAT

Episodic outbreaks of Fusarium head blight (FHB), a major fungal disease of wheat, warrant exploration of host resistance loci as a sustainable disease management strategy. A panel of 236 elite soft red winter wheat were used in greenhouse and field experiments to identify quantitative trait loci (QTL) for five FHB traits. Ghimire et al. (https://doi.org/10.1002/tpg2.20222) discovered 11 major‐effect QTL conditioning different FHB resistance types. Three QTL were expressed in multiple phenotyping environments and were considered stable and five were responsible for multiple resistance traits exhibiting pleiotropic effects. Six loci were putatively novel and could be deployed in wheat breeding programs after validation to contribute to FHB resistance.

## 9 GENOME SEQUENCE FOR THE BLUE‐FLOWERED ANDEAN SHRUB *IOCHROMA CYANEUM* REVEALS EXTENSIVE DISCORDANCE ACROSS THE BERRY CLADE OF SOLANACEAE

The tomato family (Solanaceae) has its greatest species richness in South America, with roughly a quarter of the family's diversity occuring in the Andes mountains alone. Powell et al. (https://doi.org/10.1002/tpg2.20223) sequenced the genome of *Iochroma cyaneum*, a blue‐flowered Andean shrub belonging to the tomatillo clade, to better understand the evolutionary history of the tomato family and in particular, the large and diverse berry clade, Solanoideae.  Analyses of 1355 genes from *I. cyaneum* and seven other Solanaceae genomes showed that the early branches of the Solanoideae lineage are rife with conflict. For instance, roughly equal numbers of genes support the three possible combinations of relationships among *Iochroma*, tomato, and chili pepper. This extreme disagreement among genes suggests a rapid radiation when the berry clade arose, perhaps tied to geological events such as the climate warming during the Eocene. The variation in evolutionary histories across genes will present a challenging situation for future comparative genomic studies, as, for example, there is no clear sister group for the large genus *Solanum*, which includes tomato, potato, and eggplant.

## 10 GENETIC DISSECTION OF ANTHOCYANIN PIGMENTATION IN RICE STEM

Anthocyanin pigments are secondary metabolites accumulating in aerial organs of plants including rice. Genetic dissection of purple anthocyanin pigments in rice stem was conducted using genome‐wide association study (GWAS) and whole genome resquencing (WGR). Haghi et al. (https://doi.org/10.1002/tpg2.20224) reported three genomic regions (QTLs) significantly associated to the trait; especially the relevance of a narrow interval on short arm of chromosome 6 (4.7–6.2 Mbp) was noted. Seven candidate genes in the interval were identified, including two transcription factors of bHLH and MYB types.

## 11 SUGAR TRANSPORT OF C4 CROPS DIFFERS

Although C_4_ crops possess high photosynthetic efficiency because of their carbon dioxide concentration mechanism in leaves, the productivity varies in different C_4_ species, such as maize and foxtail millet. Liu et al. (https://doi.org/10.1002/tpg2.20226) reports that not only some critical major SWEET proteins show significant upregulation in maize compared with their orthologs in foxtail millet, but also there are more quantities of maize *SWEET* genes showing high expressions than that of foxtail millet genes, suggesting that compared with foxtail millet, maize possesses higher capacity of sugar transport, the crucial determinant for crop yield and biomass. It is therefore helpful to unlock an interesting issue why some C_4_ species do not show high productivity although they have such potential.

## 12 SORGHUM GRAIN QUALITY HOTSPOTS IDENTIFIED

Sorghum breeding has been largely focused on improving grain yield overlooking grain quality that eventually affects end use. Discovering genetic loci associated with improved grain quality will help gene pyramiding and trait introgression. Ayalew et al. (https://doi.org/10.1002/tpg2.20227) identified genomic hotspots that control protein, starch, and amylose contents on chromosomes 1 and 2. These genomic hotspots collocated with DNA binding proteins that regulate starch and protein accumulation in the grain. The identified genomic regions and underlying candidate genes provide a starting point for further validation and marker‐assisted gene pyramiding to improve sorghum grain quality.

## 13 RRMEM DISCOVERS TIME‐INTERACTING QTL

The recent advancement in image‐based phenotyping platforms enables the acquisition of large amount of time‐series data for crops. Although the traditional frequentist‐based random regression model allows geneticists to investigate temporal genetic variation among individuals, it is limited to specific assumptions about the distribution of the underlying genetic variants. Qu et al. (https://doi.org/10.1002/tpg2.20228) propose a Bayesian random regression method that can implement different mixture priors for the causal variants underlying the dynamic genetic structure of time‐series data. The mixture prior in multitrait BayesC is shown as a demonstration. With biologically motivated assumptions, the proposed method achieves not only improved estimation of breeding value but also shows potential to accurately distinguish quantitative trait loci (QTL) that are invariant to time and QTL that interact with time.

## 14 IDENTIFYING AND EXPRESSION ANALYSIS OF WALNUT WD40

WD40 protein is widely involved in plant growth, development, and abiotic stress response. Walnut tree is exposed to various external environmental stimulus during its growth and development. Chen et al. (https://doi.org/10.1002/tpg2.20229) report that JrWD40 proteins were highly conserved with 4∼5 WD40 domains and *JrWD40* genes can be induced to varying degrees by low and high temperature treatments. *JrWD40‐14*, *JrWD40‐18*, *JrWD40‐34*, and *JrWD40‐3* displayed strong transcriptions response to both heat and cold stresses. Thus, *JrWD40* family genes may participate in walnut adaptation to adversity and can be used as important candidates for walnut resistance molecular breeding.

## 15 LONG‐READ‐BASED DRAFT GENOME SEQUENCE OF INDIAN BLACK GRAM IPU‐94‐1 ‘UTTARA’: INSIGHTS INTO DISEASE RESISTANCE AND SEED STORAGE PROTEIN GENES

India is the top producer of black gram. Ambreen et al. (https://doi.org/10.1002/tpg2.20234) reports a high‐quality draft genome and annotation of Indian cultivar IPU 94‐1 assembled based on long‐read sequencing data. The draft genome and annotation improves our current understanding of black gram genomics and provides fundamental resources for acceleration of its breeding programs. Black gram is an important legume crop possessing high quality seed proteins, with a potential role in achieving global food and protein security.  However, crop improvement programs in the crop to tackle low yield are hampered by lack of genomic resources. A detailed annotation of resistance genes and seed storage proteins was done providing ready resources, which enables breeders to make well‐informed decisions adding to their improvement efforts. Syntenic analysis revealed large collinear blocks between *Vigna* species reflecting conservatory patterns. In totality, the paper adds to important genomic resources required for improvement of crops belonging to Vigna complex.

## 16 GENOMIC PREDICTION IN ALFALFA USING FAMILY BULKS

Improving yield in alfalfa (*Medicago sativa* L.) can take several years as traits must be evaluated under multiple harvests. Andrade et al. (https://doi.org/10.1002/tpg2.20235) showed that genomic prediction at the family level and the use of multiple harvests data can optimize genomic prediction models for canopy height and dry matter yield. This study assessed the performance of a prediction model (GBLUP) within 11 harvests and in scenarios with multiple harvests. Within harvests, models had a predictive ability of ∼0.30 on both traits. Models had low predictive ability for future harvests. However, a substantial increase was found in scenarios that included multiple harvests in the training population to predict the average performance of a family across all harvests. The implementation of genomic prediction at family level and its performance within multiple harvests can be used as a guide for alfalfa and other perennial crops also bred in families.

## 17 THE RELEASE OF *A. CANTONIENSIS* GENOME

*Abrus cantoniensis* Hance is a native medicinal plant in southern China. Xu et al. (https://doi.org/10.1002/tpg2.20236) report the first high‐quality genome in Abrus genus, *A. cantoniensis* genome, as well as the detailed genomic information. The assembled genome size was 381.27 Mb with a scaffold N50 of 18.95 Mb, and 98.97% of the assembled sequences were anchored on 11 pseudochromosomes. This genome comprised 25,058 protein‐coding genes and 45.12% of the assemblies were repetitive sequences. Comparative genome analysis suggested that chromosome translocation and inversion played an important role in the differentiation of *Abrus*. 24 toxin‐related genes were identified, which formed two tandem gene clusters in chromosomes 2 and 3. The chromosome‐level genome of *A. cantoniensis* obtained in this work provides a valuable resource for the research in *A. cantoniensis* and *Abrus* species.

## 18 CHROMOSOME‐SCALE ASSEMBLY OF MORINGA GENOME

Chang et al. (https://doi.org/10.1002/tpg2.20238) present a chromosome‐scale assembly of the moringa (*Moringa oleifera* Lam.) genome, a highly nutritious, fast‐growing, and drought‐tolerant tree crop. Using bioinformatics tools, researchers examined the differential role of gene duplicates generated through whole genome duplication or small‐scale duplication, prominently tandem duplication, in the evolution of specific gene families. Although the former tend to be associated with transcription factor and protein modification functions, the latter are preferentially found behind the expansion of secondary metabolism gene families. Results presented here (a) support the dosage balance hypothesis to explain the reciprocal retention pattern of gene duplicates by mechanism of duplication and (b) provide a genetic roadmap to guide future breeding programs in moringa, especially those aimed at improving secondary metabolism related traits.

## 19 COMPLETE MITOCHONDRIAL GENOME SEQUENCE OF THE CULTIVATED YELLOW NUTSEDGE

Yellow nutsedge is unique in accumulating a great amount of oil in underground tubers and provides a model system for studying oil accumulation in nonseed tissues. Niu et al. (https://doi.org/10.1002/tpg2.20239) report the first complete mitochondrial genome of the cultivated yellow nutsedge. The genome contains much higher percentage of noncoding sequences and is larger in size than that in most other angiosperm plants. The gene order and clusters in the mitochondrial genome of yellow nutsedge appears to be mostly consistent with those of monocotyledons, though some species‐specific features were also observed. Phylogenetic analysis based on the shared protein‐encoding genes further demonstrate that yellow nutsedge is evolutionarily more closely related to monocotyledonary species.

## 20 PERENNIALS SURVIVE ENVIRONMENTAL CHANGES VIA TRANSCRIPTOMIC ADAPTATION

How is perennial plant sustained for tens or hundreds of years of life in the changing environment? Liu et al. (https://doi.org/10.1002/tpg2.20240) addressed this question using *Siberian larch* from Altai mountains. Gene expression profiles of *Siberian larch* from several natural populations hundred kilometers away show that environmental factors, especially temperature, dominated differential gene expression, while the contribution of genetic variation is relatively small. These results indicate that gene expression plasticity of *Siberian larch* may be the driving force of adaptation to mild environment fluctuations, and gene expression regulation is a major strategy for perennial individual plant survival during a short evolutionary history.

## 21 GENETIC AND MOLECULAR BASIS OF CLUBROOT RESISTANCE

Clubroot disease caused by *Plasmodiophora brassicae* is one of the serious threats to canola (*Brassica napus* L. subsp. *napus*) production; the development of clubroot resistant cultivars has been considered the most economic and environment friendly way of managing this disease. By using a doubled‐haploid oilseed *B. napus* population carrying genome content introgressed from rutabaga (*B. napus* L. subsp. *rapifera*), Wang et al. (https://doi.org/10.1002/tpg2.20241) identified two major loci, located at 14.41–15.44 Mb of A03 chromosome and 9.96–11.09 Mb of A08 chromosome, and their interaction confer resistance to *P. brassicae* pathotypes 3H, 3A, and 3D. Analysis of the genes from these two chromosome regions suggested that decreased expression of sugar transporter genes from A03 and increased expression of toll/interleukin‐1 receptor–nucleotide binding–leucine rich repeat genes from A08 could be the major determinant of the resistance. Single nucleotide polymorphism allele‐specific markers were developed from these two chromosome regions. Thus, the knowledge gained, and the molecular markers developed from this research could be used in breeding to develop clubroot resistant canola cultivars carrying multiple resistance genes for sustainable management of this disease.

## 22 SORGHUM'S GENETIC RESISTANCE TO ANTHRACNOSE

Anthracnose leaf blight of sorghum causes up to 50% yield loss. Khanal et al. (https://doi.org/10.1002/tpg2.20243) identified 47 significant markers, 32 candidate genes, and 47 biochemical pathways associated with resistance to anthracnose leaf blight. The molecular markers associated with resistance on chromosomes 2, 3, 5, and 10 overlap with markers identified in previous research, indicating their importance in durable anthracnose resistance. Several candidate genes have a known role in plant defense, making these primary targets for resistance breeding. These results are essential to understanding host resistance to anthracnose leaf blight and provide several targets for sorghum breeding efforts.

## 23 GENOME‐WIDE CHARACTERIZATION AND FUNCTION ANALYSIS UNCOVERED ROLES OF WHEAT LIMS IN RESPONDING TO ADVERSE STRESSES AND *TALIM8‐4D* FUNCTION AS A SUSCEPTIBLE GENE

Although LIM transcription factors play essential roles in plants, there lacks a fully understanding to wheat LIMs. Li et al. (https://doi.org/10.1002/tpg2.20246) identified 28 *TaLIM* genes from wheat, which encodes hydrophilic and unstable proteins. Expression profiling analysis showed that certain members of TaLIMs were responsive to biotic and abiotic stresses. The biological function of *TaLIM8‐4D* was analyzed and results showed that it was subcellular localization in the nucleus and could induce weak cell death in *Nicotiana benthamiana* leaves. Overexpression of *TaLIM8‐4D* could upregulate plant *pathogenesis‐related* (*PR*) genes, promoting the infection of hemibiotrophic pathogen, implying that *TaLIM8‐4D* could function as susceptible gene in the nucleus by upregulating *PR* genes and inducing cell death to promote the colonization of hemibiotrophic agent *Fusarium graminearum*.

## 24 DEEP LEARNING PREDICTS PLANT CHROMATIN

Modern molecular tools like CRISPR now allow breeders to make precise edits to a genome and have the potential to accelerate breeding in a wide range of species. However, breeders need a way to prioritize regions to edit among the potentially billions of nucleotides in a plant or animal genome. Wrightsman et al. (https://doi.org/10.1002/tpg2.20249) created a model named “a2z” that predicts which regions of any flowering plant genome are likely to be accessible or unmethylated in leaves using only genomic sequence. Mutations within accessible regions of the genome are known to be much more likely to produce significant effects on a plant's phenotype compared to the rest of the nongenic portion of the genome. a2z is most accurate on regions close to genes and accessible across many tissues. By relying solely on genomic sequence to make predictions, using a2z avoids the need to perform expensive accessibility assays and can be applied in any flowering plant species with a genome assembly.

